# Commentary: Post-traumatic benign paroxysmal positional vertigo: mechanisms, clinical phenotypes, and a structured clinical pathway for management

**DOI:** 10.3389/fneur.2026.1814393

**Published:** 2026-03-27

**Authors:** Heiko M. Rust, Rebecca M. Smith, Barry M. Seemungal

**Affiliations:** 1 Vestibular Neurology Unit, Department of Neurology, University of Basel Hospital, Basel, CH, Switzerland; 2 Faculty of Medicine, University of Basel, Basel, CH, Switzerland; 3 Centre for Vestibular Neurology, Department of Brain Sciences, Imperial College London, London, United Kingdom

**Keywords:** benign paroxysmal positional vertigo, BPPV, dizziness, traumatic brain injury (TBI) vertigo, vestibular agnosia

Kong and Seo (1) reviewed the literature on post-traumatic benign paroxysmal positional vertigo (BPPV). One of their conclusions is that modest biomechanical insults to the head may cause BPPV. We found, in acute-prospective studies, in patients (*n* = >150) with acute moderate-to-severe traumatic brain injury (TBI) [Mayo clinic classification for TBI (2)] a BPPV prevalence of up to ~60%, with BPPV being more likely the greater the forces acting upon the skull (3, 4). These findings were recently reproduced (5). On this background, the presence of BPPV could indicate the severity of the TBI to be categorized as at least moderate. As BPPV is relatively rare before the sixth decade, the presence of BPPV especially in the young might therefore serve as marker of TBI severity (6).

Kong and Seo also discuss the fact that BPPV may be overlooked or misdiagnosed in acute trauma settings. In fact, a considerable amount (c. 30%) of patients with acute TBI do not report vestibular symptoms or imbalance (7, 8) due to “vestibular agnosia” (VA). Vestibular agnosia manifests as an attenuated or absent perception of self-motion and is linked to imbalance, a condition we have reported on for the first time in TBI in an acute, prospective study (8).

The authors state that post-traumatic BPPV may differ from idiopathic BPPV in clinically meaningful ways, with which we agree, however, this difference is not considered in their proposed algorithm for treating BPPV. The algorithm is problematic since there are no controlled studies assessing the barriers to diagnosing and treating BPPV acutely (training, clinical costs, staff cognitive biases) nor data indicating when to treat and whom to treat since many patients show recurrence despite repeated BPPV particle repositioning [Calzolari et al. (8) report 40% recurrence at 6-months despite treatment at 0 and 6-months]. The authors also fail to acknowledge the lack of robust controlled data on the effectiveness of repositioning maneuvers in post-TBI BPPV. The second main issue is their recommendation that any trauma patient reporting vestibular symptoms should be assessed and treated for BPPV. Notably we reported that vestibular agnosia resulted in a 7-fold increase in missed BPPV in acute hospitalized BPPV (8) due to lack of subjective vertigo complaint, and this was replicated by Harrell et al. in a cohort of post-acute hospitalized TBI cases reporting a BPPV prevalence of 58% (*n* = 73) when all patients were assessed by examination (9) vs. 7% (10) when BPPV was screened initially by vertigo report (i.e., 10-fold difference). Our in-depth qualitative work (11) highlights that clinicians report concerns about assessing and treating post-TBI BPPV in patients who have associated neck, spinal and limb injuries, particularly in the absence of clear and specific consensus driven guidelines. It is our view that without these clinical algorithms, such as the one proposed, will be limited in their implementation.

The authors note that post-TBI may be refractory due to broader otolith dysfunction, however, the mechanism(s) mediating BPPV recurrence in TBI are as yet unclear.

Considering the still sparse knowledge on post-traumatic BPPV and the diversity of studies available, lacking comparable and high-quality data, we believe that it is premature to suggest a clinical algorithm for BPPV in TBI without controlled effectiveness studies, consensus on when it is appropriate to assess and/or treat and on whom. A feasibility study has laid the groundwork for future effectiveness trials which will also require a deeper understanding of implementation science since an effective treatment is only effective if delivering the treatment in routine care settings can be achieved.

In summary, we agree with the authors that there are critical differences between idiopathic BPPV and post-TBI BPPV, including lack of vertigo symptoms in post-TBI BPPV and its high recurrent rate. It is our view that further robust, acute, prospective controlled trials are required to determine (i) the effectiveness of repositioning maneuvers in post-TBI BPPV and (ii) the optimal time to treat (given recurrence data), as well as data and consensus informing contraindications to assessment and treatment and predictors for recurrence (to guide follow up needs) to provide a specific, implementable pathway for post-TBI BPPV assessment and treatment.
